# Protocol for targeted peripheral adeno-associated viral vector transduction of the intradental neurons in mice

**DOI:** 10.1016/j.xpro.2026.104424

**Published:** 2026-03-14

**Authors:** Ling-Yu Liu, Amy Chang, Isaac T. Berthaume, Joshua J. Emrick

**Affiliations:** 1 Department of Biologic and Materials Sciences & Prosthodontics, University of Michigan, Ann Arbor, MI 48109, USA

**Keywords:** health sciences, model organisms, neuroscience, molecular/chemical probes

## Abstract

Here, we present the methodology for surgical preparation of the mouse molar teeth and subsequent adeno-associated virus (AAV) transduction of intradental neurons within the tooth (AAV-i.d.). We describe steps for mouse positioning, microscope setup, drilling to expose pulp horns, and applying the AAV-silk mixture. We then detail procedures for composite placement to cover the preparation and trigeminal ganglion (TG) screening to visualize the surface and assess and verify AAV labeling. AAV-i.d. labels intradental neurons and traces their associated neuronal projections.

For complete details on the use and execution of this protocol, please refer to Ronan et al.^1^

## Highlights

• Workflow for novel-targeted intradental neuron transduction in the trigeminal ganglion
• Description of mouse positioning for either maxillary or mandibular molar access
• Stabilization of AAV on the tooth preparation using a silk fibroin additive
• Guidance on drilling depth, AAV-mixture application, and labeling variability

## Abstract (editor highlights)

Publisher’s note: Undertaking any experimental protocol requires adherence to local institutional guidelines for laboratory safety and ethics.

## Graphical abstract

**Figure:**
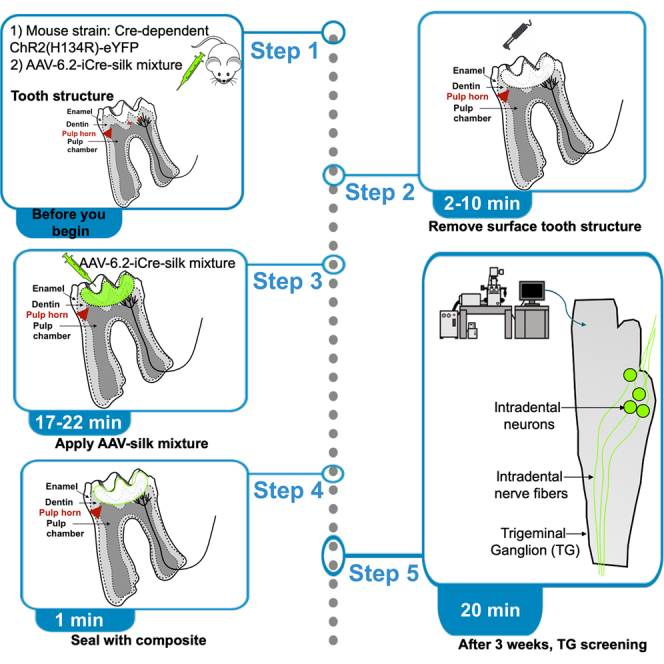

## Before you begin

The first successful labeling of neurons innervating the inner tooth (i.e., intradental neurons) was reported in 1956, utilizing silver impregnation.^2^ Over subsequent decades, a variety of chemical tracers have been developed and employed for neuron labeling in dental and sensory research. These include **h**orse**r**adish **p**eroxidase (**HRP**),^3^^,^^4^ HRP conjugated with **w**heat **g**erm **a**gglutinin (**HRP-WGA**),^5^ Fluorogold,^6^ Dil and DiO,^7^ non-fluorophore-conjugated **c**holera **t**oxin subunit **B** (non-fluor. **CTB**),^8^^,^^9^ and more recently, fluorophore-conjugated CTB (fluor. **CTB**).^10^^,^^11^^,^^12^

In parallel, **a**deno-**a**ssociated **v**irus (**AAV**) systems have gained prominence as powerful, flexible tools for transducing neurons, offering high efficiency, long-term labeling, and reduced toxicity for long-term studies. Notably, cell-type-specific capsid selection strategies such as the **Cre** recombinase-based **A**AV **t**argeted **e**volution (CREATE) platform, introduced by Dr. Deverman et al.,^13^ and further optimized by Dr. Chan et al.,^14^ have revolutionized the specificity and efficiency of neuronal targeting through modified capsid proteins. Specifically, AAV6.2 – also referred to as AAV6(F129L) – has been shown to transduce airway epithelium, muscle and lung tissues, as described by Dr. Limberis et al., and Dr. van Lieshout et al.,.^15^^,^^16^ For a detailed comparison of these labeling methods including their discovery dates, molecular types, efficiency, specificity, and tissue applications please refer to Table 1 below.

**Table 1:** Comparison of existing methods for labeling efficiency and specificity

Discovery year	Method	Labels cell body or fibers	Injection & labeling time	Labeling efficiency	CNS/Sensory labeling (tooth?)	Species	Imaging resolution for fibers	Specificity	Toxicity
1956	Silver impregnation^2^	Fibers (mainly); some cell bodies	Variable, slow (hours–days after fixation)	Moderate	Sensory (Tooth: Yes)	Human	LM, moderate	Moderate, not cell-type specific	Low (can be harsh fixatives)
1981, 1984	HRP^3^^,^^4^	Cell bodies & fibers (mainly retrograde)	Pulp Injection: hours; Labeling: 2–6 days	High	CNS & Sensory (Tooth: Yes)	Cat, Rat	LM (can combine with EM), moderate	Good, but may have non-specific uptake	Mild, but can induce reactions
1988	HRP-WGA^5^	Cell bodies & fibers (anterograde and retrograde)	Pulp Injection: hours; Labeling: 2–5 days	High	CNS & Sensory (Tooth: Yes)	Rat	LM (can combine with EM), moderate	Good, but may have non-specific uptake	Mild, but can induce reactions
1989	Fluorogold^6^	Cell bodies (strong), some fibers	Pulp Injection; Labeling: 2–8 days	High	Sensory (Tooth: Yes)	Rat	LM, moderate; visible at cell level	High (retrograde)	Generally low; can be cytotoxic at high doses
1989	Dil and diO^7^	Both cell bodies and fibers (antero/retrograde)	Incubations; Labeling: at least two weeks for the distance of 5mm (slow)	High (diffuses in lipid memb.)	CNS & Sensory (Tooth: variable)	Many	LM, high; especially with confocal	Moderate (non-specific membrane label)	Low
1988, 2007	Non-fluor. CTB^8^^,^^9^	Cell bodies & fibers (retrograde)	Tissue Injection: variable; Labeling: 1–14 days	High	CNS & Sensory (Tooth: Not directly)	Rat, Cat	LM & EM, moderate-high	High (retrograde)	Low
2009, 2020	Fluor. CTB^10^^,^^11^^,^^12^	Cell bodies & some fibers	Tissue Injection: variable; Labeling: 3–8 days	Very High	CNS & Sensory (Tooth: Yes^12^)	Rat, Mouse	LM, high with confocal	High (retrograde, multi-color tracing possible)	Very low
1997	AAV-GFP^17^	Cell bodies & fibers (GFP expression)	Cervical spinal cord Injection; Expression: 1–4 weeks	High	CNS & some sensory	Rat	High (depends on reporter)	Cell specificity via promoter	Very low
2010	AAV^18^(multiple serotypes)	Both (gene expression chosen cell type)	DRG Injection; Expression: 1–4 weeks	Moderate–High (serotype-dependent)	CNS & Sensory (Tooth: not in this study)	Rat	High (fluorescence/confocal/2-photon)	Can be cell-type specific (promoter-dependent)	Very low (AAV highly safe)
2009, 2018	AAV6.2^15^^,^^16^	Cell bodies (transduction marker)	Culture Injection: 1–4 weeks for expression	High (improved over AAV6)	CNS/Sensory (not directly tooth)	Mouse	High (immunofluorescence)	High (can add cell specificity via promoter)	Very low
2016	rAAV2-retro^19^	Projection neurons (cell bodies via retrograde transport)	Tissue Injection; Labeling: 2–4 weeks	Very High	CNS, Sensory (tool for both; retrograde access, tooth not shown)	Mouse	Very high (can use clearing/confocal)	Extremely high (retrograde; projection neurons)	Very low
2017	Engineered AAVs (AAV-PHP.eB and AAV-PHP.S,^14^	Both, broad (PHP.eB: CNS & PHP.S: SNS)	Intravenous Injection; Labeling: 2–4 weeks	Very High	CNS & Sensory (broad, incl. DRG)	Mouse	Very high (cellular–fiber, in vivo)	Very high (can target DRG, PNS)	Very low
2025	AAV6.2^1^	Cell bodies & fibers (YFP expression)	Tooth Injection; Labeling: 2–4 weeks	Very High	Sensory (Tooth: Yes)	Mouse	Very high (cellular–fiber	Very high	Very low

HRP, **H**orse**r**adish **p**eroxidase; HRP-WGA, HRP conjugated with **w**heat **g**erm **a**gglutinin; Dil, [1,1′-dioctadecyl-3,3,3′,3′-tetramethylindo-carbocyanine perchlorate; diI-C∼8-(3)] and DiO, [3,3′- dioctadecyloxa-carbocyanine perchlorate; diO-C∼8- (3)] (the carbocyanine dyes); Non-fluor. CTB, non-fluorophore-conjugated **c**holera **t**oxin subunit **B** (CTB); Fluor.CTB, fluorophore-conjugated CTB; AAV, **a**deno-**a**ssociated **v**irus (AAV); DRG, **D**orsal **r**oot **g**anglion; CNS, **C**entral **n**ervous **s**ystem; PNS, **P**eripheral **n**ervous **s**ystem; LM, **L**ight **m**icroscopy; EM, **E**lectron **m**icroscopy.

To label intradental neurons, we initially selected the AAV6.2 serotype for tooth injections based on its demonstrated success in the transduction of peripheral tissue cells. We now report that AAV6.2 achieves transduction and labeling of intradental neurons. We did not validate alternative serotypes such as rAAV2-retro and AAV-PHP.S^14^^,^^19^ that have been reported to transduce peripheral sensory neurons. The injection and labeling times, as well as labeling efficiencies for each serotype, are summarized in Table 1 for comparison. We did not perform the direct comparison of different labeling methods using AAV-i.d.

**Figure 1:**
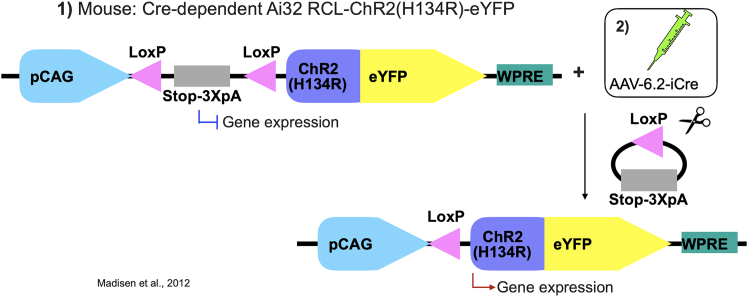
The Cre-LoxP labeling strategy The viral vector carrying AAV-6.2-iCre results in the expression of Cre to mediate site-specific DNA recombination at two LoxP sites, excision of the stop cassette, and subsequent cell type-specific expression of ChR2(H134R)-eYFP.^20^

### The Cre-LoxP labeling strategy

1. Select and obtain animals of the appropriate Cre driver mouse strain and adeno associated viral vectors (AAVs) for your experiment. The combination will induce expression of a construct in the desired intradental neurons.

**CRITICAL:** To selectively target the intradental neurons *Scn10a*-Cre or *S100b*-Cre driver lines will be used based on their known transcriptional expression of *Scn10a* and *S100b*.^1^^,^^12^

**CRITICAL:** To visualize intradental neurons in *Scn10a-*Cre/*S100b-*Cre mouse strains, a Cre dependent AAV that produces GFP expression would be used. (i.e., AAV6.2-dlox-eGFP).

**CRITICAL:** If the AAV is Cre dependent, a Cre driver mouse strain must be used, otherwise, neuronal labeling will fail. Cre dependent AAVs are usually denoted as “dlox,” “flex,” “DIO,” or “floxed.”

***Note:*** An alternative strategy^20^ (Figure 1) to label intradental neurons independent of their expression of *Scn10a* or *S100b*, a Cre-dependent reporter mouse strain such as Ai32 RCL-ChR2(H134R)/eYFP would be paired with AAV6.2-iCre (AAV6.2-hEF1a-iCre). Here, the AAV provides the necessary Cre element for expression in the Cre-dependent reporter mouse strain.

***Note:*** Generally, the choice of Cre driver mouse strain depends on the known transcriptomic expression in your target cell population. Cre driver strain availability and commercial sources can be found at Mouse Genome Informatics (MGI). Desired Cre driver mouse strains can also be designed and engineered independently.

***Note:*** Adeno associated viral vectors (AAVs) are selected based on their dependence on Cre recombinase and desired payload (e.g., fluorescent proteins, effector proteins). AAVs are available from commercial vendors (e.g., ETH Zurich VVF, Addgene), but can also be produced by core facilities and in house.

### Reagent preparation

**Timing: 10 min (steps 2–4)**

2. Attach black braided suture to the lateral retractors.
3. Prepare silk fibroin (50 mg/mL) aliquots of 50 μL and store in the −80C.
4. Prepare AAV6.2-iCre (7.2×10E12 vg/mL) aliquots of 2.5 μL and store in the −80C.

### Preparation of AAV-silk mixture

**Timing: 5 min (steps 5–6)**

5. Thaw aliquots of 2.5 μL of silk fibroin (50 mg/mL) and AAV6.2-iCre (7.2×10E12 vg/mL) on ice.
6. Combine equal volumes of the viral vector AAV6.2-iCre with silk fibroin at a 1:1 ratio.
  a. Pipette silk fibroin into viral vector.
  b. Pipette up and down to gently mix.
  c. Spin down mixture with a mini centrifuge at max. speed RCF 2,000 × *g*.

***Note:*** Addition of silk fibroin creates a viscous solution that concentrates, contains, and localizes viral vector during tooth application.

***Note:*** In our experiments, 2.5 μL of AAVs when combined 1:1 with 2.5 μL of silk fibroin will be adequate for transduction of up to 2 molar teeth.

***Note:*** Thawed silk fibroin can be stored at 4C and remains stable for 1 month. Thawed AAVs can be stored at 4°C and remain stable for 1 week.

**CRITICAL:** Make sure the silk fibroin and viral vector are mixed well and spun down, keep the AAV-silk mixture on ice.

### Innovation

Prior work to study the sensory neurons innervating the inner tooth primarily relied on tooth pulp exposure and biochemical labeling. While these methods achieved good labeling of the cell bodies of neurons within the trigeminal ganglia, they required removal of all overlying dentin, labeled neurons broadly, and did not enable visualization of additional cellular structures at distant sites (i.e., neuron projections). This protocol presents the methodology for judicious surgical preparation of mouse molar teeth and the novel application of adeno-associated viral vector (AAV) transduction of intradental neurons within the tooth. Here we detail the use of a biochemical additive, silk fibroin,^21^ to stabilize the AAVs and facilitate transduction of the mineralized tooth. Our technique spares portions of the overlying dentin where intradental neuron projections reside.^1^ In conjunction with the Cre-LoxP system, **AAV-i.d.** enables simultaneous, cell-type specific, genetic targeting of intradental neurons. Furthermore, the induction of fluorescent labeling using **AAV-i.d.** enables visualization of both their soma and their associated fiber which will contribute to further understanding of the neural basis and circuits for reflexes and pain that originates with the intradental neurons. This approach can be adapted with various Cre-LoxP combinations (or alternative recombinases) or alternative craniofacial structures to achieve cell-type specificity for trigeminal sensory neurons of interests.

### Institutional permissions

Users of this protocol must obtain required institutional approvals for the use of animals before beginning. All animal experiments described were conducted in accordance with protocols approved by the University of Michigan Institutional Animal Care and Use Committee following NIH guidelines.

## Key resources table

**Table:** 

REAGENT or RESOURCE	SOURCE	IDENTIFIER
**Bacterial and virus strains**		

Viral vector: AAV6.2-iCre (7.2×10E12 vg/mL)	ETH Zurich- Viral Vector Facility	v225-6(F129L)-ssAAV-6(F129L)/2-hEF1a-iCre-WPRE-bGHp(A)

**Chemicals, peptides, and recombinant proteins**		

Silk Fibroin (50 mg/mL)	Advanced BioMatrix	Cat. #5154
Isofluorane	Vetone	13985-528-60
Flowable Composite	Pentron	N11VA
Ophthalmic ointment	Fisher Scientific	NC0490117

**Experimental models: organisms/strains**		

Mouse strain: Cre-dependent Ai32 RCL-ChR2(H134R)/eYFP, 6–14 week, male and/or female	Jackson Lab	#012569

**Software and algorithms**		

Digital Camera Software	Amscope	v.10.11.2024

**Other**		

Olympus SZX7 Stereo Dissecting microscope with boom stand and tilting adapter	Olympus	S/N 2205180929
Confocal Microscope	Olympus	FV3000
Light Stand	N/A	Emrick Lab
Nose Cone Stand	N/A	Emrick Lab
Microscope Digital Camera	Amscope	MU1003
SomnoSuite® Low-Flow Anesthesia System for Mice and Rats	Kent Scientific	SS-01
Tooth Drill	Delanie Electric Nail Drill	C20 (BK-A08)
¼ round Carbide Burs	House Brand Dentistry	HSB-401152
Retraction cord (yarn for retracting lower Incisors)	N/A	Emrick Lab
Lateral oral retractors, blunt tips, 2.5 mm	Kent Scientific	SURGI-5016-2
Black braided silk suture	MDmaxx	SP116
Gel loading pipette tip, 0.2 mm flat	Costar	Cat. No. 4815
Eppendorf Research Plus Pipette-0.1–2.5 mL	Eppendorf	Cat # 3123000012
Woodpecker LED.B Wireless High Power Blue LED Curing Light	Net32/Ebay	K192233
Endodontic paper points	Dentsply	P07679
Surgical tweezers	Fine Scientific Tools	11923–13
Mini Centrifuge Max. Speed RCF 2,000 × *g*	Fischer Scientific	75-004-061
**Custom Stereotax**		
Breadboard	Thorlabs	MB1015/M
Posts (M6 × 1.0 screw)	Thorlabs	HW-KIT2/M M6X1.0 Hardware Kits
Suspension wire (from a #2 Butterfly Clamp)	Acco	344134
Laboratory tape	Grainger	846EK6

## Step-by-step method details

### Mouse positioning and microscope setup

**Timing: 15–30 min: 5–10 min (steps 1–10); 10–20 min (steps 11–13)**

In this section, we describe how to enable oral tissue isolation to visualize the maxillary molars and mandibular molars using two different setups of the stereomicroscope. While multiple experimenters would aid in carrying out steps within this protocol, we routinely carry out the protocol with a single person.

**Figure 2:**
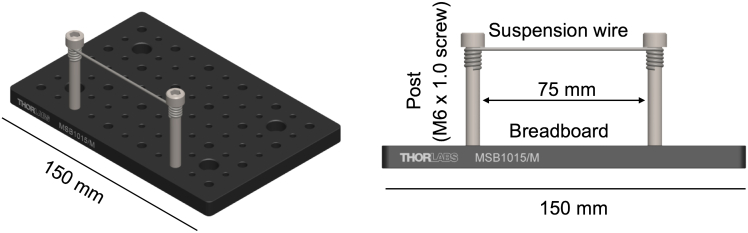
Assembled stereotax used for oral isolation and visualization of mouse molar teeth CAD model from isometric projection (left) and front view (right). Suspension wire connects two posts that are attached to the breadboard. Scale bar, 150 mm.

1. Assemble stereotax (See Figure 2).
  a. Screw two posts (M6 × 1.0 screws) into the breadboard spaced 75 mm apart.
  b. Straighten #2 butterfly clamp to create suspension wire.
  c. Wrap ends of suspension wire around each post.
  d. Suspension wire height can be adjusted by sliding ends up or down the posts.
2. Set up the isoflurane machine for initial induction: 475 mL/min at 4.5%.
3. Place the mouse in the chamber for initial induction.

**CRITICAL:** Wait for at least 3 min, or at least one minute after the mouse is under anesthesia and no longer demonstrated the righting reflex.

4. Apply ophthalmic ointment to the eyes of the mouse.
5. Place mouse on breadboard.

**Figure 3:**
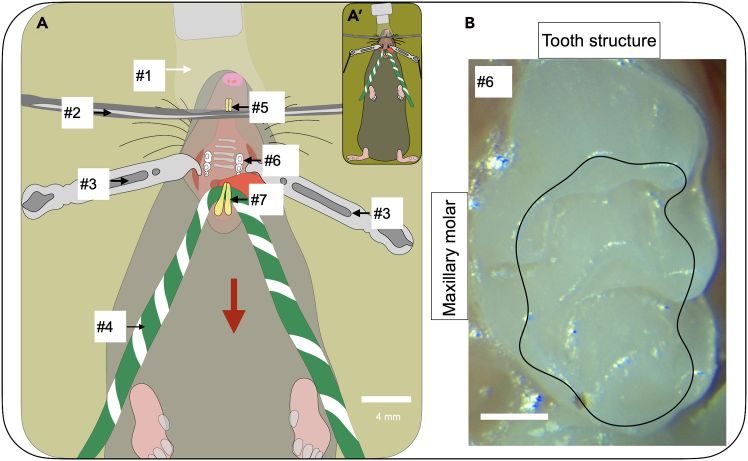
The maxillary (max.) molars are visualized in a supine mouse (A) Schematic figure shows the mouse in supine position. The setup is designed to open the mouth for optimal visualization of the max. molars. The upper incisors (#5) are secured over the suspension wire (#2), while the jaw is opened upward using retraction cord (#4: green and white yarn) tied around the lower incisors (#7). Two lateral oral retractors (#3) placed on either cheek enables visualization of the max. molars. Scale bar, 4 mm. Numbers denote the following: #1: Nose cone; #2: Suspension wire; #3: Lateral oral retractor; #4: Retraction cord; #5: Upper Incisors; #6: Maxillary molar M1; #7: Lower Incisors. (B) The max. molar tooth structure. Black outline represents future preparation borders. Scale bar, 0.4 mm.

***Note:*** For maxillary molars the mouse is in a supine position with its head facing away from operator. (See Figure 3 for the mouse supine position).

**Figure 4:**
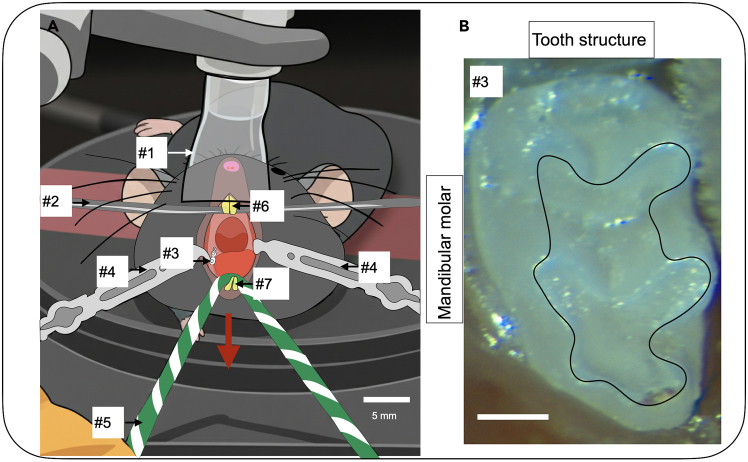
The mandibular (mand.) molars are visualized in a prone mouse (A) Schematic figure shows the mouse in prone position. The setup is designed to open the mouth for optimal visualization of the mand. molars. The upper incisors (#6) are secured over the suspension wire (#2), while the jaw is opened downward using retraction cord (#5: green and white yarn) tied around the lower incisors (#7). Two lateral oral retractors (#4) placed on either cheek enables visualization of the mand. molars. Scale bar, 5 mm. Numbers denote the following: #1: Nose cone; #2: Suspension wire; #3: Mandibular molar M1; #4: Lateral oral retractor; #5: Retraction cord; #6: Upper Incisors; #7: Lower Incisors. (B) The mand. molar tooth structure. Black outline represents future preparation borders. Scale bar, 0.4 mm.

For mandibular molars the mouse is in a **prone** position with its head facing toward operator.

**Figure 5:**
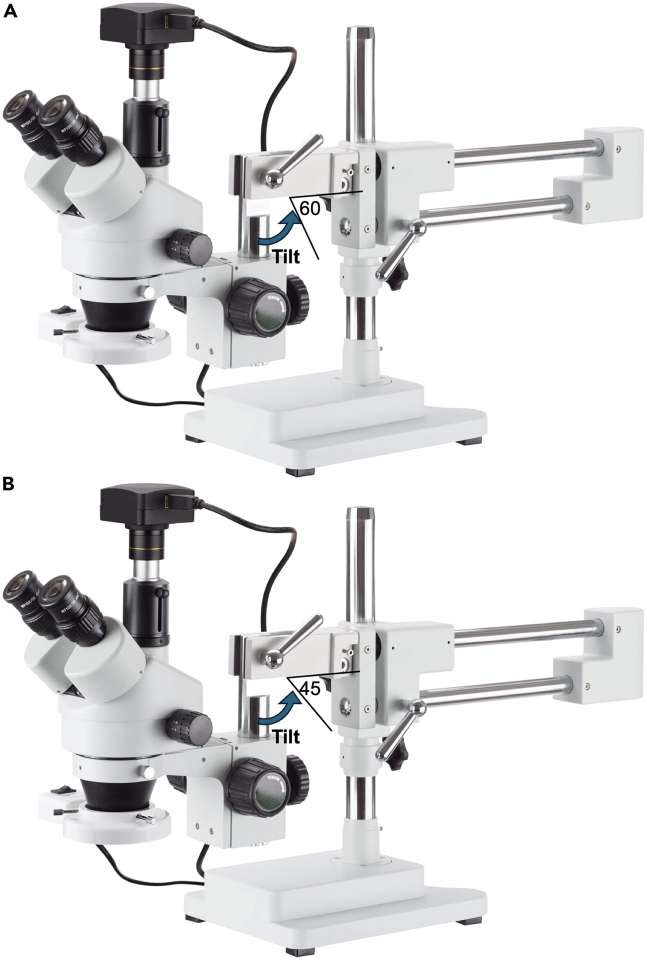
Example stereoscope with forward tilt capability (A) Tilt should be adjusted to 60° from horizontal; (B) Tilt should be adjusted to 45° from horizontal. Photograph of a generic stereoscope with body and tilt that will enable visualization of both maxillary and mandibular molars. Blue arrow indicates forward tilt away from the operator. As depicted, stereoscope body is vertical (=90° from horizontal).

(See Figure 4 for the mouse **prone** position).

6. Adhere the upper incisors to the suspension wire to hold the mouse in place. (See Figures 3 #5 and 4 #6).
  a. Place a 0.5–1 mm diameter bead of flowable composite on the upper incisors.
  b. Light cure the composite for 5 s.
7. Switch the isoflurane machine from induction to nose cone.
8. Place the mouse with the visual field of the stereoscope.

**CRITICAL:** Ensure that the nose cone fits over the nose without obstructing air flow. (See Figures 3 #1 and 4 #1).

9. Administer isoflurane using the nose cone for maintenance of anesthesia at 175 mL/min at 2.0%.

***Note:*** Isoflurane can be adjusted to 0.7%–3.4% based on mouse respiration.

10. Monitor and maintain the animal’s body temperature at 36°C.
11. Retract the jaw using retraction cord (yarn) tied around the lower incisors. (See Figures 3 #4 and 4 #5). Troubleshooting 1.

***Note:*** Secure the retraction cord to the stereotax base or surrounding work surface using laboratory tape.

**CRITICAL:** For maxillary molars, pull the retraction cord more horizontally toward the operator away from the bar (See Figure 3, red arrow).

**CRITICAL:** For mandibular molars, pull the retraction cord is pulled immediately downward from the bar (See Figure 4, red arrow).

12. Use the lateral oral retractors (2 blunt tips, 2.5 mm) to increase the width of access to the oral cavity. (See Figure 3 #3 and 4 #4). Troubleshooting 1.
  a. Pull the black braided suture connected to the lateral oral retractor laterally away from the mouse.
  b. Use laboratory tape to secure the lateral oral retractor position.

**CRITICAL:** Place the lateral oral retractors into the buccal vestibule immediately lateral to the molars to ensure isolation of the molars from the mucosa. (See Figure 3 #3 and 4 #4 for placement of the lateral oral retractors).

***Note:*** Re-position the lateral oral retractors and re-secure suture with tape sequentially. Repeat until the molars are isolated and easily visualized.

13. Use the stereoscope to ensure visual access to the target molars. Troubleshooting 1.
  a. Visualization of maxillary molars: Adjust the forward tilt (See Figure 5A) of the microscope body away from the operator to 60° from horizontal.
  b. Visualization of mandibular molars: Adjust the forward tilt (See Figure 5B) of the microscope body away from the operator more to 45° from horizontal.

**CRITICAL:** Stereoscope setup will require different viewing angles for observing the maxillary molars or mandibular molars to accommodate the anatomical differences. (See Figures 5A and 5B).

**CRITICAL:** Monitor breathing of the mouse following molar access. If the mouse is gasping the isoflurane should be lowered.

### Drilling to expose pulp horns

**Timing: 4–12 min: 2 min (step 14); 2–10 min/per tooth (step 15)**

In this section, we describe how to use the drill and identify the exposure of pulp horns. In Ronan et al.,^1^ we showed that the intradental nerve fibers penetrate into one-third of the dentin from pulp chamber. To allow the AAV-silk mixture to reach the maximum number of healthy, undamaged nerve endings, we carefully expose only the pulp horns. We expect that the AAV-silk mixture does not reach the nerve endings if the preparation is too shallow. Alternatively, we expect that more nerve endings are damaged when the preparation is too deep. Either of these contribute to reduced (or absent) neuronal labeling.

14. Prepare the drill.
  a. Ensure a new ¼ round bur is attached to the drill handpiece.
  b. Set the drill speed to maximum speed (35,000 RPM).
  c. Check that the drill is set to “Forward” setting.
15. Remove the coronal cusps of the mouse molar using the bur. Troubleshooting 2, 3, and 4.
  a. Cut with the bur at a 45° to horizontal remove the structure from the occlusal surface of the tooth.
  b. Remove 200–300 μm to expose the pulp horns of the mouse molar (at least two pulp horns are exposed).

**Figure 6:**
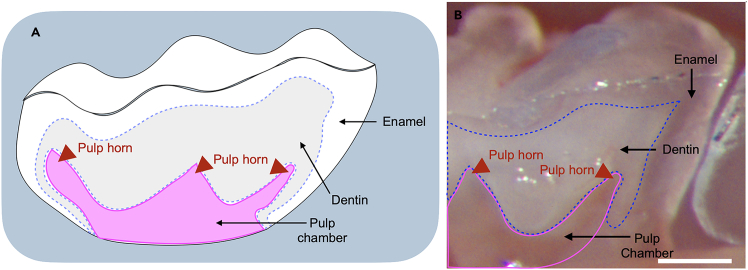
An example molar dissected and hemisected along the longitudinal axis (A) Schematic figure shows the hemisected molar along the longitudinal axis. (B) The blue dashed line outlines the dentin, while the magenta line outlines the pulp chamber. The arrows indicate the enamel (transparent), dentin (translucent), and pulp chamber from superficial to deep within the tooth. The red arrowheads indicate the pulp horns. Scale bar, 0.2 mm.

**Figure 7:**
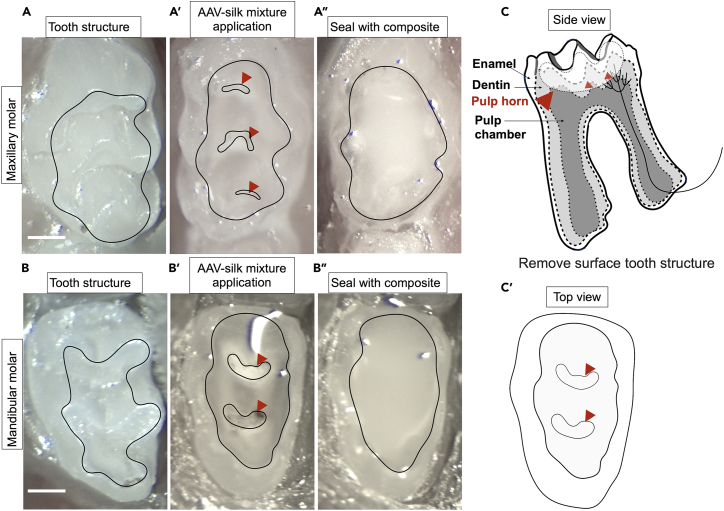
Representative images of the exposed pulp horns in the maxillary and mandibular first molar The pulp horns are exposed in the maxillary first molar (A-A″) and the mandibular first molar (B-B″). (A, B) The molar before drilling. (A′, B′) the pulp horns exposed and applied with AAV-silk mixture during application. (A″, B″) After application, the molar is sealed with composite. Black outline indicates the preparation border. (C) Schematic figure shows the hemisected molar along the longitudinal axis with the indications of enamel, dentin, pulp chamber, and preparation depth and area. (C′) Schematic figure shows the top view of the drill area and the exposed pulp horns. The red arrowheads indicate the pulp horns. Scale bar, 0.4 mm.

**CRITICAL:** Since no cooling is used during this step, apply only light downward pressure while cutting to avoid excessive heating of the tooth. Use visual cues to identify if the pulp horns have been exposed. At least two pulp horns should be exposed. Do not expose the entire pulp chamber (Figure 6, 7A′, 7B′ and 7C′, the pulp horns are indicated by red arrowheads). See Figures 7A’, 7B′ and 7C′ for drill depth and preparation area on maxillary and mandibular molar. Any bleeding from the pulp horn should be minimal, if it does bleed, please use endodontic paper points to remove any blood and wait until the bleeding has stopped. Drilling shape should be concave, deepest at the buccal-lingual midline, and tapering to the tooth surface at the edges of the occlusal surface (See Figure 7 for the surface outline of the tooth).

***Note:*** Make sure the drill depth exposes at least two pulp horns (See Figures 7A’, 7B′, and 7C′ for drill depth on maxillary and mandibular molar and the pulp horns are indicated by red arrowheads). We typically expose three pulp horns on maxillary molars and two pulp horns on mandibular molars. If the drill depth is too shallow or too deep, the neuron labeling is less efficient or absent. (Please see Figures 10 and 11). The total time for drill per molar is 2–10 min depending on experience. The location and shape of the preparation within the occlusal surface enable focused AAV-silk application to the preparation and prevent its unwanted spread to surrounding tissues.

### Application of AAV-silk mixture

**Timing: 17–22 min: 2 min (step 16); 15–20 min (steps 17–19)**

In this section, we describe how to apply AAV-silk mixture to the exposure of pulp horns.

16. Draw up 2 μl AAV-silk mixture with a 2 μl pipette and flat gel loading tip, 0.2 mm flat.

**CRITICAL:** The AAV-silk mixture is viscous and may be challenging to draw up into the pipette tip initially. Ejecting past the second stop then drawing up the mixture can help with initial loading.

17. Pipette the AAV-silk mixture into the preparation until it reaches capacity. Troubleshooting 5.

**CRITICAL:** Ensure that it does not overflow onto any adjacent structures (mucosa, other teeth, etc.). If any solution spreads, remove it with endodontic paper points immediately.

***Note:*** The AAV-silk mixture can be added to the adjacent molar in the arch if it has been drilled within this timeframe.

18. Air dry AAV-silk mixture gently using a pipette tip connected to vacuum suction.

**CRITICAL:** Do not accidentally suction the AAV-silk mixture. Re-apply before the AAV-silk mixture completely dries. When there is a thin layer of moist solution remaining, stop drying and replenish the mixture immediately.

***Note:*** We visually monitor the shape of the drop of AAV-silk mixture after application to evaluate the drying process. We use the surface light reflection to monitor the surface shape as it transitions from convex (outward) to concave (inward).

***Note:*** Repeated drying and application will concentrate the AAV concentration.

19. Repeat 10 times of application and drying over 15 min.

**CRITICAL:** Avoid complete desiccation of AAV-silk mixture to prevent damage of AAV particles.

***Note:*** The site is continuously incubated with the AAV-silk mixture for 15–20 min to facilitate virus penetration and transduction. Every drying period after application is approximately 1.5 min.

### Composite placement to cover preparation

**Timing: 1 min (steps 20**–**25)**

In this section, we describe how to cover the preparation and pulp horns with dental composite.

20. Eject 0.5–1 mm diameter bead of dental composite onto a working surface.
21. Use forceps to pick up an endodontic paper point.
22. Collect a small amount of flowable composite (approximately the size of the head of a pin) on the tip of the endodontic paper point.
23. Cover the preparation with the composite. (See Figure 7A″ and 7B″ for the surface of the tooth).
24. Remove any excess composite using an endodontic paper point to prevent overloading of restoration material.
25. Light cure the composite for 10 s.

**CRITICAL:** Keep the curing light adequate distance from the tooth and composite to prevent heating during curing.

### Completion of the procedure

**Timing: 25 min: 5 min (steps 26–31); 20 min (step 32)**

In this section, we describe the process of releasing the mouse after the procedure. After three weeks, trigeminal ganglion (TG) screening is performed to visualize the surface of the TG, and assess and verify AAV labeling.

26. Turn off anesthesia.
27. Gently remove the retractors from the mouse.
28. Detach retraction cord from mandibular incisors.
29. Detach composite from upper incisors.
30. Remove the nose cone.
31. Ensure that the mouse is awake and recovered in a clean cage before placing back into cage.
32. Verify AAV labeling efficiency after three weeks post-application.

**Figure 8:**
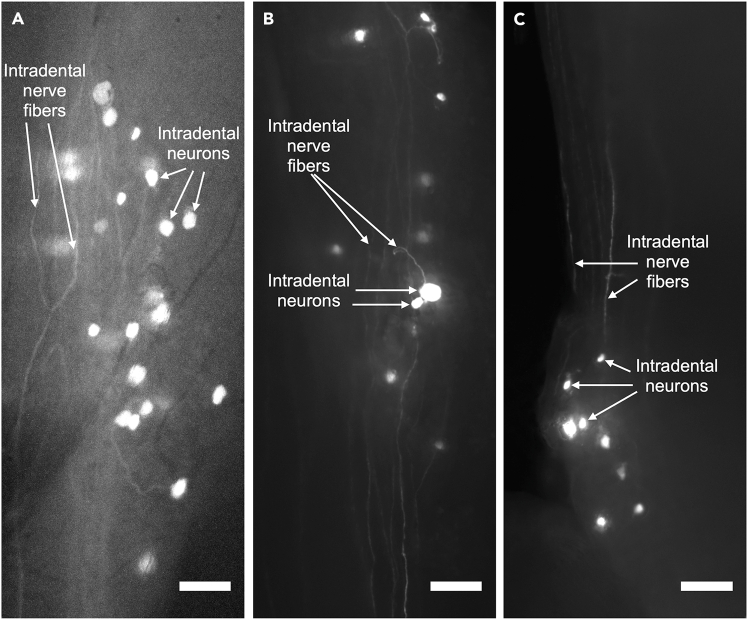
AAV-i.d. labeling of intradental neuron cell bodies and fibers (A–C) Representative micrographs captured 3 weeks after transduction of neurons using AAV-i.d. and induction of fluorescent reporter expression. (A) Labeling identified at the dorsal surface of the trigeminal ganglion within the skull. (B and C) Labeling identified at either the (B) dorsal or (C) ventral surface of a dissected, isolated trigeminal ganglion. Intradental neuron cell bodies (white) and intradental nerve fibers (gray) are labeled as indicated. Scale bars, 100 μm.

**CRITICAL:** Three weeks post-application, trigeminal ganglion (TG) screening is performed to visualize the surface of the TG, and assess and verify AAV labeling efficiency. Expression may be present before 3 weeks, but this should be empirically determined. (See Figure 8 for examples of expected labeling of intradental neurons and nerve fibers).

## Expected outcomes

This protocol outlines a novel workflow for transduction of intradental neurons using viral vectors that necessitates exposure pulp horn, but not complete exposure of the dental pulp. The targeted neuron labeling enables visualization of nerve fibers and cell bodies using a fluorescent reporter and confocal microscopy. Both the dorsal and ventral trigeminal ganglia (TG) are expected to exhibit strong positive labeling. The dorsal surface of the TG demonstrates labeled nerve fibers and cell bodies from this procedure are illustrated in Figure 8. Although not discussed in detail here, this protocol would achieve labeling of associated central projections of the intradental neurons in the brain region. Furthermore, this protocol could be adapted to other craniofacial and mineralized tissues with trigeminal innervation.

## Quantification and statistical analysis

Transcriptomic classification utilizes *in situ* hybridization to identify intradental neurons.^1^^,^^12^^,^^22^ Labeled neurons identified using **AAV-i.d.** are primarily C6 neurons found in the trigeminal ganglion. Single sections of the trigeminal ganglion were collected from Ai32 (Cre-dependent ChR2-YFP) mice (n=4) receiving AAV-Cre tooth injections. Sections were processed for in situ hybridization for *YFP*, *Scn10a*, and *S100b*. Of 123 total labeled YFP+ intradental neurons, 103 were positive for *Scn10a* and *S100b*+^1^(Ronan et al., Figure S5A and S5B).

## Limitations

Our approach necessitates minimal pulp horn exposure to achieve transduction of the intradental nerve terminals within the dentin. Pulp exposure will affect the local environment of the dental pulp and would be expected to produce an inflammatory response and possible infection. Following this procedure, intradental neurons were found within the trigeminal ganglion after 3 weeks indicating that the procedure does not result in loss of labeled intradental neurons. Intradental neurons captured using this protocol were found to elicit digastric muscle contractions as measured by electromyography (EMG) when activated using optogenetics. While this single critical function was preserved, the long-term consequence of pulp exposure and any effect on the intradental neurons is not known.

## Troubleshooting

### Problem 1

Inability to observe and access the molar. Related to Step 11, 12, and 13 [step-by-step method details].

Potential Causes: The oral cavity does not appear to be opened sufficiently wide. This is likely due to a loosened retraction cord (See Figures 3, #4 and 4, #5) and/or in sufficient tension in the lateral oral retractors (See Figures 3, #3 and 4, #4).

### Potential solution

• Return to Step 11 and re-adjust the lab tape to ensure that the retraction cord is firmly secured to the workbench, resulting in adequate mouth opening.
• Return to Step 12 and re-adjust the lab tape to ensure that the lateral oral retractors are tightly fixed to the workbench, allowing the mouth to open sufficiently wide.

### Problem 2

Difficulty in visualizing the pulp horns in the molar. Related to Step 13 and 15 [step-by-step method details].

**Figure 9:**
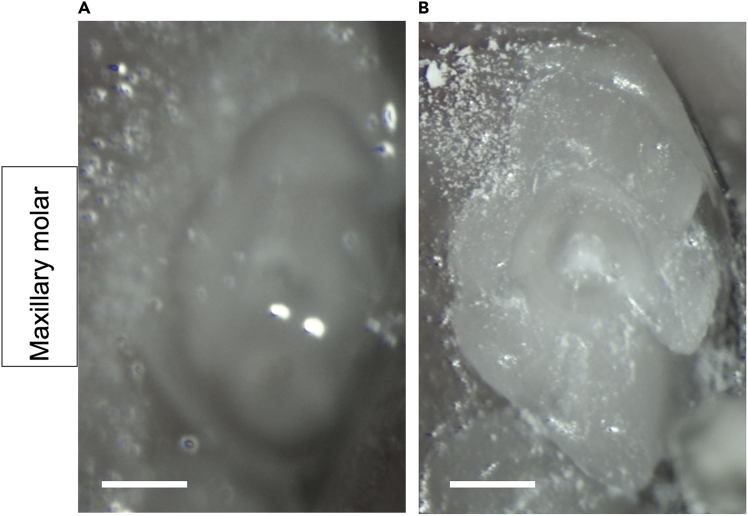
Troubleshooting failure to visualize pulp horns Images of a maxillary molar M1 that is (A) out of focus or (B) in focus with pulp horns visible. Scale bar, 0.4 mm. Related to Troubleshooting 2.

Potential Causes: Failure to achieve proper focus on the molar. See Figure 9.

### Potential solution

• Ensure the stereoscope is properly focused on the molar. See Step 13 and Figure 9B.
• Follow the guidance for exposing pulp horns. In the graphical abstract, the pulp horn is indicated by a red arrowhead. Exposed pulp horns can also be seen in Figure 6, as well as Figures 7A’ and 7B’. To expose the pulp horn in the molar, drill to a depth of approximately 200–300 μm. See Figures 11D and 11E for more examples of successful mandibular molar labeling. See Figure 11F for successful maxillary molar labeling.

### Problem 3

**Figure 10:**
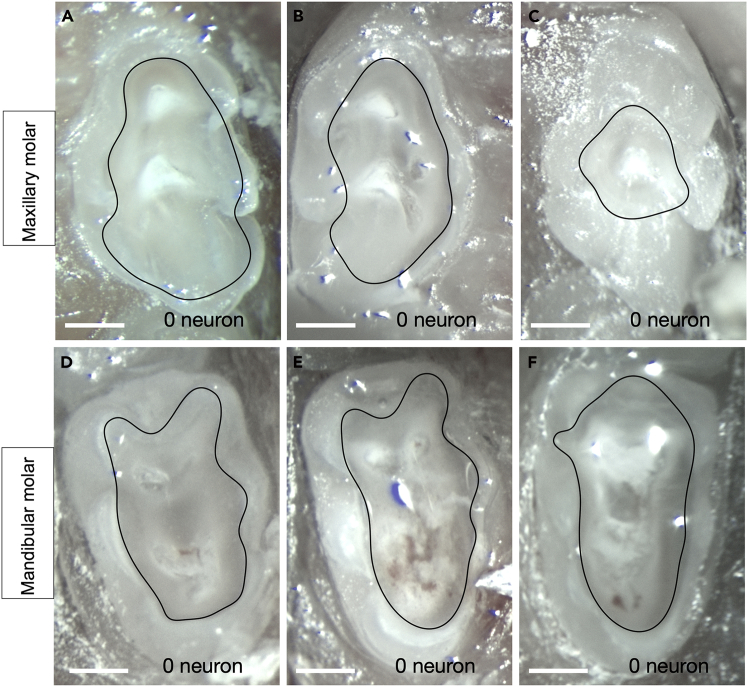
Troubleshooting failure in intradental neuron labeling due to preparation issues Images of maxillary molars featuring shallow drilling (A–C) and mandibular molars featuring deep drilling (D–F). All examples corresponded with a lack of labeling. Black outline indicates the preparation border. Scale bar, 0.4 mm. Related to Troubleshooting 3.

Failure in intradental neuron labeling due to issues with prepartion. Related to Step 15 [step-by-step method details]. See Figures 10A–10C for maxillary molars, and Figures 10D–10F for mandibular molars.

Potential Causes: Drill depth is either too shallow or too deep.

### Potential solution

**Figure 11:**
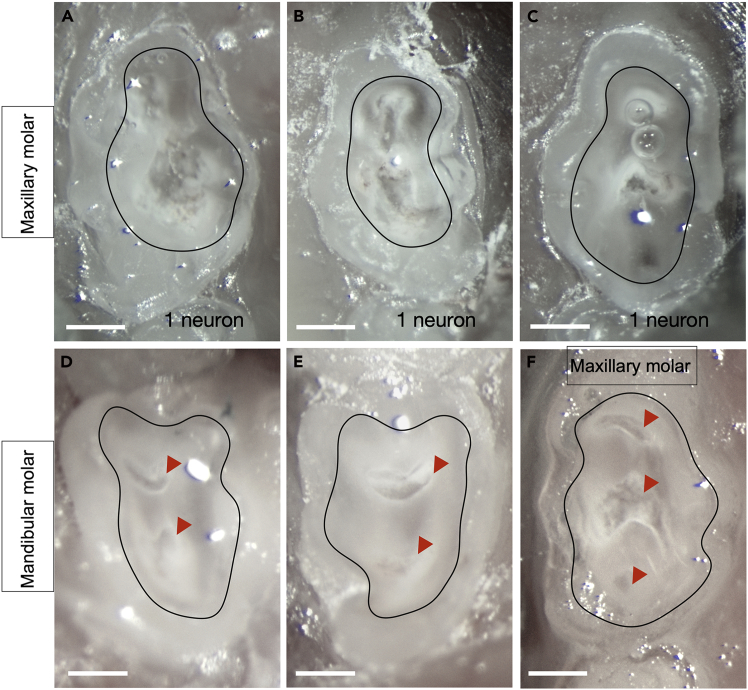
More examples of molar tooth preparations for AAV-i.d. labeling Images of maxillary molars featuring deep drilling (A–C) that corresponded with few cell bodies being labeled (1 cell). Images of (D–E) mandibular and (F) maxillary molar preparations that corresponded with successful labeling. Black outline indicates the preparation border. Scale bar, 0.4 mm.
• Ensure the mixing ratio of AAV to silk fibroin is at least 1:1 to prevent rapid desiccation of the viral vector upon application to the tooth occlusal surface.

• Follow the guidance for exposing pulp horns. In the graphical abstract, the pulp horn is indicated by a red arrowhead. Exposed pulp horns can also be seen in Figure 6, as well as Figures 7A’ and 7B’. To expose the pulp horn in the molar, drill to a depth of approximately 200–300 μm. See Figures 11D and 11E for more examples of successful mandibular molar labeling. See Figure 11F for successful maxillary molar labeling.

### Problem 4

Few cell bodies labeled. Related to Step 15 [step-by-step method details]. See Figures 11A–11C.

Potential Causes: Excessive drilling pressure, excessive drilling depth, and/or prolonged drilling time.

### Potential solution

• Set the drill speed at 35,000 RPM and avoid generating excessive heat. Use a light touch, applying light downward pressure when removing enamel and dentin.
• Drill only until the pulp horn is reached. Exposing the pulp chamber may injure nerves, resulting in fewer visible cell bodies. Bleeding should be minimal, and drilling should cease as soon as the pulp horns are exposed.
• Limit drilling time to <8 min. Our experiments showed that drilling longer than 8 min negatively impacts AAV uptake.

### Problem 5

AAV-silk mixture does not remain within the preparation and overflows onto adjacent structures during the 15 min of application. Related to Step 17 [step-by-step method details].

Potential Causes: The mouse’s head is positioned too high for the maxillary molars and/or the preparation is too shallow. Excessive AAV-silk mixture is added to the preparation cavity.

### Potential solution

• Drill depth should extend beyond the enamel and enter the dentin-pulp junction. If the drill depth is too shallow, the AAV-silk mixture will not be retained and may compromise labeling visualization.
• For maxillary molar drilling, position the mouse supine with head tilted back. For mandibular molar drilling, the occlusal table should be parallel to the work surface.
• Apply only enough AAV-silk mixture to fill the prepared cavity. Application of AAV-silk mixture should proceed slowly to avoid overfilling and spilling out.

## Resource availability

### Lead contact

Further information and requests for resources and reagents should be directed to and will be fulfilled by the lead contact, Joshua J. Emrick (jjemrick@umich.edu).

### Technical contact

Further information on executing this protocol should be directed to and will be answered by the technical contact, Ling-Yu Liu (liuly@umich).

### Materials availability

Materials used in this protocol are listed in the key resources table.

### Data and code availability

All data reported in this paper will be shared by the lead contact upon request.

## Acknowledgments

This work was supported by K22 DE029779 and R01 DE032345 (to J.J.E.).

## Author contributions

L.-Y.L. and J.J.E. conceptualized and developed this protocol with assistance from A.C. L.-Y.L., A.C., I.T.B., and J.J.E. performed AAV-i.d. experiments. L.-Y.L., A.C., and J.J.E. drafted and finalized the manuscript. All authors gave approval of the final version of the manuscript.

## Declaration of interests

The authors declare no competing interests.
